# Annotations

**Published:** 1892-02-13

**Authors:** 


### Annotations.

**A Veteran of the Sunday Fund.**

Sir Rutherford Alcock has felt compelled, through increasing years and infirmities, to sever his connection with the active work of the Hospital Sunday Fund. Although no man can grudge Sir Rutherford his late and well-earned leisure, all readers of newspapers and others who take interest in public men will feel a pang of regret that so resolute and kindly a veteran should at length have had to submit to the inevitable. Most of all will Sir Rutherford's colleagues on the Council of the Sunday Fund feel the loss of his presence and of his experienced wisdom and earnest co-operation. Sir Rutherford Alcock is now an old man. He was born in 1809, and cannot be therefore less than 83. He obtained his medical qualification in 1831, long before most of us were born. He spent some years on the medical staff of the British auxiliary forces in Spain and Portugal and rapidly gained distinction in both services. In 1837 he had risen to the rank of Deputy-Inspector General of Hospitals, and later received Her Majesty's permission to wear the Insignia of the Tower of Portugal, the Cross of Charles III., and the Commanders' Cross of the Order of Isabella II. of Spain, conferred upon him for his services in the field. Sir Rutherford has rendered many public services to his country; but it is chiefly of his connection with hospitals and their adjuncts that we desire here to speak. He has been an active member of the Council of the Hospital Sunday Fund for many years. He has taken a keen interest in all subjects connected with the improvement of hospital administration. When the question of establishing pay hospitals and pay wards was first opened, he gave to it the advantage of his great wisdom and experience, and his connection with the movement had much to do with its after success. Sir Rutherford also aided in the formation of the Hospitals Association; and, indeed, every question connected with hospital management, finances, and usefulness has found in him a steadfast supporter and a capable helper. Such services as Sir Rutherford has rendered receive comparatively little recognition at the hands of the public. But as we have before pointed out in these columns, the voluntary hospitals of London and the country constitute one of the most important of all the factors of our modern social system. The money which they annually raise, and which they spend for the most part so admirably, amounts to several millions: is. indeed, equal to the total revenues of some of the smaller States. When men like Sir Rutherford Alcock come forward to manage these important affairs, and manage them so generally well that adverse critics can find little or no fault with them, we cannot but feel that a deep debt of gratitude is owed by the whole community for such self-denying, capable, but unrewarded public services. Sir Rutherford Alcock retires from the Hospital Sunday Fund with all men's regard and all men's regret.

**Is Influenza Infectious?**

There is a strong consensus of opinion in England in favour of the doctrine that influenza is an infectious disease. This doctrine is by no means held by all competent physicians. Sir Douglas Maclagan, a famous professor in the University of Edinburgh, and a man of ripe years and experience, belongs to the class of philosophic doubters, though he admits that so long as the question is in such a condition of uncertainty he would isolate influenza patients exactly as he would patients suffering from admittedly infectious diseases. Sir Douglas says: "Notwithstanding the strong and important statement of Doctors Parsons and Buchanan and others, I have still my doubts as to its spreading by infection in the ordinary sense. But as this is a matter of doubt, and I fancy that professional opinion is in favour of the theory of infection, it is better to err on the safe side and practise isolation. I know, however, of no facts which would enable me to say how long the isolation should be maintained. The excessive prostration which follows the attack generally, I think, keeps the patient long enough in the sick room to give him time to be safe enough." It is refreshing to see even one solitary opinion which differs from that of the great majority of medical men in this country. The common experience is that when one or two leaders in medicine, or well-known microscopists, have expressed their judgment, all the other members of the profession utter pious "Amens," and there the matter ends. This is melancholy! All spirit seems to be dying out among the great mass of doctors. There are absolutely no convincing grounds for attributing any kind of practical infallibility to any medical scientist living or dead. The case of Koch ought of itself to be sufficient to generate in the ordinary medical mind a critical faculty even if such a faculty were non-existent before. It was miraculous to see how all the medical world, both small and great, flocked to Berlin at a mere wave of the magician's wand. But what is the result to science and medicine of Koch's cure of consumption theory? Whatever may be the result to science, the result to medicine is a serious impairment of its credit in the eyes of all judicial opinion outside the ranks of professional men. We are, it seems, to have a scientific enquiry into the nature, causes, and treatment of influenza. We must take care that the bacteriologists do not get it all their own way. A bacteriologist is seldom a physician; he is a dissector, a cultivator, and a microscopist. But dissections, cultivations, and microscopic investigations have often very little to do with clinical medicine. They are important as aids; and the compositor who sets up types is important as an aid to the dissemination of an author's views. It would, however, be a somewhat risky experiment to give the compositor precedence of the author; and it is a very risky experiment indeed to make the bacteriologist the leading authority in practical medicine, as opposed to the bedside physician.

**The Registration of Nurses.**

The Royal British Nurses' Association is about to make application for a Royal Charter. All who are interested will heartily welcome the fact. In making its application the Association must define its position and objects, and the means whereby it hopes to give effect to its aims. Its pretentions must be submitted to the Privy Council, a tribunal which is at once judicial and public. The Privy Council will determine whether the British Nurses' Association is a representative body; and whether it is in accordance with public policy that that Association shall be endowed with the dignity and authority which it claims. Not only will the Association have the opportunity of stating its own claims, but the Midwives' Institute and the Associated Nurse Training Schools, which have practically created the nursing world as it now exists, will be asked to give their experienced judgment on the question of registration. By this means the whole subject will be discussed in all its bearings by impartial and judicial minds, and settled finally. Those who know are aware that an efficient system of Nurse Registration is already in force. It has often been demonstrated, and it will no doubt be convincingly proved before the Privy Council, that each Nurse Training School keeps an adequate register of all the nurses it trains, and that these combined registers are the best, and, indeed, the only trustworthy records which the public can ever obtain. The Board of Trade has already considered the B.N.A.'s scheme of Registration and rejected it. When the B.N.A. applied for incorporation, the reply was that, "After careful consideration, the Board of Trade are unable to satisfy themselves that the means which the Association propose to adopt are either adequate to carry out their object satisfactorily or so free from objection as to warrant the Board of Trade in the issue of a license." Before its application to the Board of Trade the B. N. A. drafted a Royal Charter, a copy of which we have, for submission to the Privy Council, and publicly announced the fact. But when, apparently, it was found that success was then hopeless on the merits of the case alone the proceedings ceased. Her Royal Highness the Princess Christian, who is the embodiment of kind heartedness and loyalty to the cause of women, is, it is said, about to press the claims of the B. N. A. personally upon the Queen in Council. It is difficult to believe that the more judicial members of the Council of the British Nurses' Association can have given their consent to such a course. Public policy alone can determine the claims of the Association. But, if the published statements are correct, the most constitutional monarch in the world, is to be asked to decide a question of public policy by methods which are distinctly unconstitutional; and a Royal Princess is to be the standard bearer in this unprecendented enterprise. Can they be called friends either of the Princess Christian, the Royal Family, or Her Majesty's Government who permit themselves to stand sponsors for methods so questionable as these? There can be little doubt, from the grounds on which the decision of the Board of Trade, and the united opposition of the Nurse Training Schools rest, as well as from the inherent weakness of the non-representative foundations on which the British Nurses' Association relies, that its case on the merits is fore-doomed to failure.

